# Supplementary material for: Comprehensive Annotation and Functional Exploration of MicroRNAs in Lettuce
Source: Front Plant Sci. 2021 Dec 24;12:781836. doi: 10.3389/fpls.2021.781836 (PMC8739914; doi:10.3389/fpls.2021.781836)
Supplement: Supplementary file 3 [file Data_Sheet_9.ZIP › GSM2230753.plot/Lsa-miR160b_Lsat_1_v5_gn_2_100181.1_2076_TPlot.pdf]

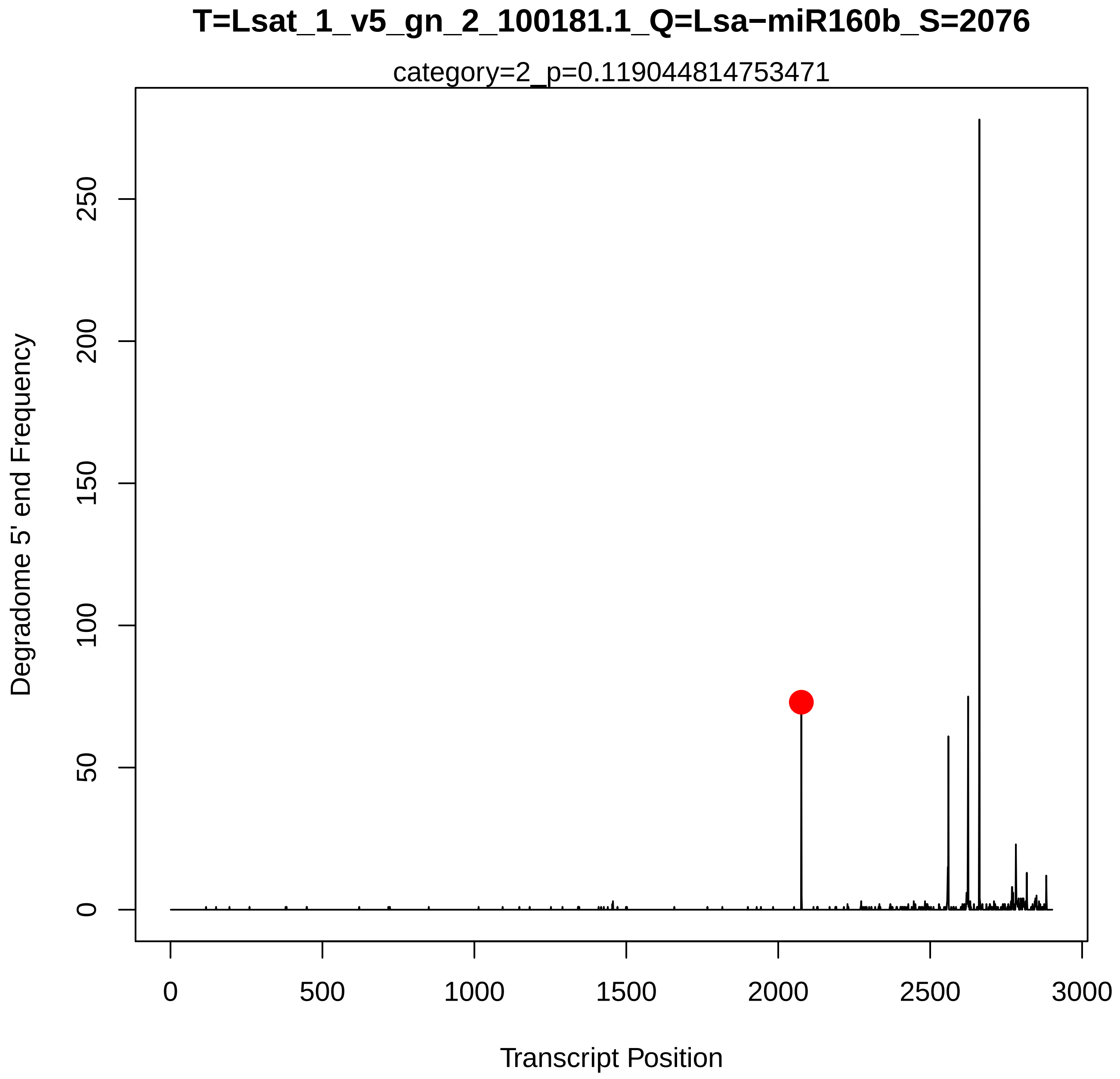
T=Lsat_1_v5_gn_2_100181.1_Q=Lsa−miR160b_S=2076
category=2_p=0.119044814753471
Degradome 5' end Frequency
0
50
100
150
200
250
0
500
1000
1500
2000
2500
3000
Transcript Position
